# Acute effect of daily fine particulate matter pollution on cerebrovascular mortality in Shanghai, China: a population-based time series study

**DOI:** 10.1007/s11356-019-05689-8

**Published:** 2019-07-01

**Authors:** Khadija Akter Leepe, Mei Li, Xin Fang, Ayako Hiyoshi, Yang Cao

**Affiliations:** 1 Department of Applied Statistics, School of Business, Örebro University, Örebro, Sweden; 2 Center for Assessment of Medical Technology in Örebro, Örebro University Hospital, Örebro, Sweden; 3 Unit of Biostatistics, Institute of Environmental Medicine, Karolinska Institutet, Solna, Sweden; 4 Clinical Epidemiology and Biostatistics, School of Medical Sciences, Örebro University, Örebro, Sweden; 5 Department of Public Health Sciences, Stockholm University, Stockholm, Sweden

**Keywords:** Fine particulate matter, Cerebrovascular mortality, Generalized additive model, Multicollinearity, Principal component analysis, Shrinkage smoother, Least absolute shrinkage and selection operator

## Abstract

Numerous studies have investigated the impacts of ambient fine particulate matter (PM_2.5_) on human health. In this study, we examined the association of daily PM_2.5_ concentrations with the number of deaths for the cerebrovascular disease on the same day, using the generalized additive model (GAM) controlling for temporal trend and meteorological variables. We used the data between 2012 and 2014 from Shanghai, China, where the adverse health effects of PM_2.5_ have been of particular concern. Three different approaches (principal component analysis, shrinkage smoothers, and the least absolute shrinkage and selection operator regularization) were used in GAM to handle multicollinear meteorological variables. Our results indicate that the average daily concentration of PM_2.5_ in Shanghai was high, 55 μg/m^3^, with an average daily death for cerebrovascular disease (CVD) of 62. There was 1.7% raised cerebrovascular disease deaths per 10 μg/m^3^ increase in PM_2.5_ concentration in the unadjusted model. However, PM_2.5_ concentration was no longer associated with CVD deaths after controlling for meteorological variables. The results were consistent in the three modelling techniques that we used. As a large number of people are exposed to air pollution, further investigation with longer time period including individual-level information is needed to examine the association.

## Introduction

Air pollution represents the biggest environmental risk to health, and outdoor air pollution alone has been associated with an estimated three million premature deaths in a recent year (World Health Organization (WHO), 2016). Harmful substances, such as carbon monoxide, nitrogen dioxide, and heavy metals from natural and unnatural origins, including volcanoes to industrial activities, vehicle emission, and combustion of fossil fuel, can take the forms of solid particles, liquid droplets, or gasses (Fiordelisi et al. 2017). Fine particulate matter with a size less than or equal to 2.5 μm in diameters (PM_2.5_) is the sum of such solid or liquid pollutants suspended in the air and particularly important as it tends to stay longer in the air because of its small size (Lin et al. 2018), increasing the chance of inhalation.

PM_2.5_ has been shown to have significant adverse effects on human health, including reduced lung function and increased risk of chronic bronchitis, heart disease, lung cancer, and various forms of cardiovascular and cerebrovascular diseases and mortality (Fiordelisi et al. 2017; Lu et al. 2015). Mechanisms linking PM_2.5_ and human health outcomes have not been fully understood, but unlike larger particles, small particles like PM_2.5_ can penetrate deep into the respiratory system or absorbed into the bloodstream and may increase systemic inflammation and alter autonomic nervous system activity (Fiordelisi et al. 2017; Shah et al. 2015).

Although air quality has been improved over the years, many cities and regions in European and Asian countries still suffer from PM_2.5_ concentration higher than 10 μg/m^3^ on an annual average, above the limit recommended by the WHO (Thunis et al. 2017). An increase in PM_2.5_ concentration by 10 μg/m^3^ was associated with 8–27% higher lung cancer risk (Pope et al. 2002; Turner et al. 2011) and 4% higher all-cause mortality risk (Pope et al. 2002), while a reduction by 10 μg/m^3^ expands life expectancy for 0.35 years (Correia et al. 2013). Emergency hospital admissions for cerebrovascular disease increased by 1.3% for every ≥ 10 μg/m^3^ increase in PM_2.5_ concentration (Leiva et al. 2013).

The aim of our study was to evaluate the acute effect of PM_2.5_ pollution on cerebrovascular disease (CVD) mortality using data from Shanghai, China, between 2012 and 2014. We focused on the influence of PM_2.5_ concentration on death during the same day. As we adjusted for a number of meteorological variables and some of them show multicollinearity, the secondary aim was to examine the impact of the difference of adjustment techniques that are commonly used in the presence of collinearity: principal component analysis (PCA), shrinkage smoother, the least absolute shrinkage and selection operator (LASSO) methods.

## Materials and methods

### Study setting and data

The data used this study include daily observations on the number of CVD deaths, PM_2.5_ concentrations, and meteorological conditions between 2012 and 2014 in Shanghai, China, with a total of 1091 observed days. During the study period, the population of Shanghai was approximately 24.0 million, and the life expectancy was around 83 years (Shanghai Statistics Bureau 2014). The detailed description of the data was published elsewhere (Fang et al. 2017). Briefly, daily average PM_2.5_ concentrations between January 1, 2012, and December 31, 2014, were obtained from the air quality monitoring station of the US Consulate General in Shanghai and the Shanghai Meteorological Bureau. Only the measurements from a single air quality monitor station were available during the study period. The daily mortality data during the corresponding time period for all the 16 administrative districts in Shanghai were obtained from the Causes of Death Registry of Shanghai Municipal Centre for Disease Control and Prevention. The causes of death were coded according to the International Disease Classification Codes, version 10, and the codes for cerebrovascular diseases deaths were I60–I69. Thirteen meteorological variables and the day of the week were used as potential confounding factors.

### Statistical model

Mean, standard deviation (SD), median, the first quartile (Q1), and the third quartile (Q3) were calculated for daily CVD deaths, PM_2.5_ concentrations, and meteorological variables. Univariate associations between CVD mortality and PM_2.5_ and meteorological variables were evaluated using a generalized linear model with a log link function. Generalized additive model (GAM) was used for multivariate analysis. The GAM is a generalization of the generalized linear model and is widely used in time series studies on health effects of air pollution because it does not expect a particular functional form of a relationship, and is flexible for modelling nonlinear associations (Crawley 2013). It exploits the quantity of a response variable *Y* from a given distribution with different independent variables by estimating non-parametric functions of the independent variables, which are connected to the response variable through a link function. Because of the nonlinear relationship between death counts and weather conditions, and exponential form of daily death counts within a fixed period of time, the effect of PM_2.5_ on daily deaths in the present study was modelled as:

$$ \mathrm{Log}\left[E(Y)\right]=\mathrm{Log}\left[{\mu}_t\right]={\beta}_0+{\beta}_1{X}_t+{\sum}_{i=1}^n{f}_i\left({Z}_i\right)+{\beta}_j{D}_j $$

$$ \mathrm{and}\ Y\sim \mathrm{Poisson}\ \left({\mu}_t\right) $$

where *E*(*Y*) is the expected mean of deaths on day *t*, *β*_0_ and *β*_1_ are the intercept and slope term respectively, *X*_*t*_ is the PM_2.5_ concentration on a day *t*, *f* is the smoothing function, *Z*_*i*_ is the confounding variables (i.e., time and weather conditions), and *D*_*j*_ is the dummy variables of the day of the week (DOW) and *j* = 1, 2, … , 6.

The smoothing functions *f*_*i*_ are composed of non-parametric splines of confounding variables. Splines are polynomial curves that are connected at inner knots. The widely used splines in GAM are defined as the natural spline, cubic spline, or B-spline with a pre-specified number of knots. In our study, two types of smoothing splines, cubic regression spline (*cr*) and thin plate spline (*tp*), were used and compared. To avoid over-fitting, for each of confounding variables of time and weather conditions, the optimal number of knots (*k*) was selected based on Akaike’s information criterion values using iterative processes. We compared *k* = 4, 5, 6, 7, and 8 because most of the studies show that *k* = 3 is sufficient, and often there was no notable improvement with more than 8 knots.

The coefficient of PM_2.5_ multiplying by 10 is interpreted as a percentage increase in cerebrovascular deaths per 10 μg/m^3^ increment in PM_2.5_ concentration.

### Controlling for multicollinearity

Except for controlling for temporal trends in daily cerebrovascular deaths, meteorological variables were considered as potential confounding factors (Jimenez-Conde et al. 2008). Multicollinearity among 13 meteorological variables was examined using the Spearman correlation and variance inflation factor (VIF), and statistically significant multicollinearity was found among the variables. To deal with the multicollinearity and high dimensionality of these confounding variables, we used three different approaches. The first approach used the principal component analysis (PCA) method to convert the variables into mutually orthogonal principal components (PCs) (Yang et al. 2015). Because the reduction of the number of variables, or dimensionality, on the other hand, may lead to a loss of useful information about the original data, we also repeated the analysis using shrinkage smoothers as an alternative approach. Shrinkage is the procedure of compressing extreme values towards a central value (Tibshirani 1996). The benefit of using the shrinkage smoothers is to shrink less important variables into zero (Marra and Wood 2011). When using PCA and shrinkage techniques, we applied both *cr* and *tp* splines. The third approach used the least absolute shrinkage and selection operator (LASSO) regularization, which selected variables using a penalty *α* = 1 (Friedman et al. 2010).

All analyses were conducted in R version 3.5.1 (R Foundation for Statistical Computing, Vienna, Austria) using packages *psych*, *mgcv*, and *glmnet*. Two-sided *p* values less than 0.05 are considered statistically significant.

## Results

### Descriptive statistics and univariate association

The descriptive statistics of daily mortality caused by cerebrovascular diseases, PM_2.5_ concentrations, and the meteorological variables are presented in Table 1. The mean of daily PM_2.5_ concentration is 55 μg/m^3^. The daily average deaths for the cerebrovascular diseases are 62 over the study period. The estimated coefficient with PM_2.5_ is 1.653 × 10^−3^, corresponding to a 1.653% increment in daily CVD deaths per 10 μg/m^3^ increment in PM_2.5_ concentration. All meteorological variables other than extreme wind speed showed a statistically significant association, and higher value tended to be associated with lower death risk apart from the daily atmospheric pressure variables.

**Table 1:** Descriptive statistics and univariate association for the risk of cerebrovascular disease death

Variable	Mean	SD	Median	Q1	Q3	*β*	SE	*p* value
CVD deaths	62	14	60	51	71			
PM_2.5_ (μg/m^3^)	55	39	46	29	69	1.653 × 10^−3^	9.219 × 10^−5^	< 0.001
PreAvg (kPa)	101.61	0.88	101.51	100.82	102.26	0.1576	0.0045	< 0.001
PreMax (kPa)	101.80	0.90	101.84	101.01	102.49	0.1586	0.044	< 0.001
PreMin (kPa)	101.34	0.87	101.35	100.58	103.67	0.1531	0.0046	< 0.001
TemAvg (°C)	17	9	18	9	24	− 0.0172	0.0004	< 0.001
TemMax (°C)	21	9	22	13	28	− 0.0160	0.0004	< 0.001
TemMin (°C)	14	9	15	6	22	− 0.0168	0.0004	< 0.001
HumAvg (%)	70	13	72	62	79	− 0.0011	0.0003	< 0.001
HumMin (%)	51	18	51	38	63	− 0.0011	0.0002	< 0.001
RainVol (mm)	33	104	0	0	11	− 0.0002	4.055 × 10^−5^	< 0.001
Windspd (m/s)	2.8	1.0	2.7	2.1	3.4	− 0.0187	0.0041	< 0.001
WindMax (m/s)	5.2	1.2	5.0	4.3	5.8	− 0.0066	0.0032	0.0419
WindExt (m/s)	8.6	2.2	8.2	7.0	9.8	− 0.0019	0.0017	0.278
Sunshine (h)	4.7	4.0	4.8	0	8.2	− 0.0048	0.0010	< 0.001

CVD deaths stands for the deaths for cerebrovascular diseases. PreAvg, PreMax, and PreMin stand for the daily average, maximum, and minimum atmospheric pressure, respectively. TemAvg, TemMax, and TemMin stand for the daily average, maximum, and minimum temperature, respectively. HumAvg and HumMin stand for the daily average and minimum humidity measured as percentage. RainVol stands for the daily rain volume. Windspd, WindMax, and WindExt are daily average, maximum, and extreme wind speed, respectively. SD stands for standard deviation. Q1 and Q3 stand for the first and third quartiles. *β* stands for regression coefficient for the variable in generalized linear regression model, accompanied by SE (standard error) and *p* value

There was multicollinearity among PM_2.5_ and meteorological variables (Table 2). Most of the correlation coefficients are statistically significant. For example, daily average atmospheric pressure has a highly negative relationship with the average temperature (*r* = − 0.87), average humidity is negatively correlated with the sunshine (*r* = − 0.67), and volume of rain is negatively correlated with the sunshine (*r* = − 0.61). According to the VIF values, daily average pressure, maximum pressure, minimum pressure, daily average temperature, maximum temperature, minimum temperature, daily average humidity, minimum humidity, maximum wind speed, and extreme wind speed are highly correlated to other variables and daily average rain volume, wind speed, and sunshine are moderately correlated to other variables (Table 3) (O’Brien 2007). For example, the VIF value of daily average pressure indicates that the variance of the estimated coefficient of the variable is inflated by a factor of 273.34 as it is highly correlated with at least one predictor variable. The correlations between PM_2.5_ and meteorological variables are rather weak, and most of them have a Spearman’s *r* < 0.30 (Table 3).

**Table 2:** Spearman correlation coefficients between the meteorological variables

	PreAvg	PreMin	PreMax	TemAvg	TemMin	TemMax	HumAvg	HumMin	RainVol	Windspd	WindMax	WindExt	Sunshine
PM_2.5_	0.24*	0.24*	0.23*	− 0.33*	− 0.37*	− 0.26*	− 0.08*	− 0.23*	− 0.15*	− 0.34*	− 0.21*	− 0.22*	− 0.01
PreAvg		0.98*	0.98*	− 0.87*	− 0.87*	0.85*	− 0.25*	− 0.25*	− 0.2*	− 0.1*	− 0.1*	− 0.04	− 0.01*
PreMin			0.96*	− 0.85*	− 0.84*	− 0.82*	− 0.26*	− 0.25*	− 0.2*	− 0.1*	− 0.16*	− − 0.09*	0.001
PreMax				− 0.89*	− 0.89*	− 0.84*	− 0.24*	− 0.24*	− 0.1*	− 0.08	− 0.09*	− 0.01	− 0.04
TemAvg					0.98*	0.98*	0.09*	0.12*	0.03	0.10*	0.09*	0.03	0.18*
TemMin						0.98*	0.18*	0.24*	0.11*	0.14*	0.09*	0.05	0.07
TemMax							0.007	8 × 10^−4^	− 0.04	0.07	0.09*	0.01	0.29*
HumAvg								0.90*	0.61*	− 0.09*	− 0.12*	− 0.10*	− 0.67*
HumMin									0.59*	0.05	− 0.03	− 0.005	− 0.72*
RainVol										0.06	0.10	0.14*	− 0.61*
Windspd											0.75*	0.71*	0.04
WindMax												0.90*	0.08*
WindExt													0.02

PresAvg, PreMax, and PreMin stand for the daily average, maximum, and minimum atmospheric pressure, respectively. TemAvg, TemMax, and TemMin stand for the daily average, maximum, and minimum temperature, respectively. Humvg and HumMin stand for the daily average and minimum humidity measured as a percentage. RainVol stands for the daily rain volume. Windspd, WindMax, and WindExt are daily average, maximum, and extreme wind speed, respectively. *p < 0.05

**Table 3:** Variance inflation factor (VIF) of meteorological conditions

Variable	VIF
PreAvg	273.34
PreMax	97.96
PreMin	82.04
TemAvg	308.86
TemMax	118.51
TemMin	89.96
HumAvg	7.66
HumMin	10.97
Rainvol	1.30
Windspd	3.04
WindMax	6.52
WindExt	5.43
Sunshine	2.78

PreAvg, PreMax, and PreMin stand for the daily average, maximum, and minimum atmospheric pressure, respectively. TemAvg, TemMax, and TemMin stand for the daily average, maximum, and minimum temperature, respectively. HumAvg and HumMin stand for the daily average and minimum humidity measured as a percentage. RainVol stands for the daily rain volume. Windspd, WindMax, and WindExt are daily average, maximum, and extreme wind speed, respectively

### Results of the GAM-PCA approach

The first four principal components derived from PCA explained approximately 91% variation of the original data (Table 4). After adjusting for meteorological conditions and day of the week, there was no longer a statistically significant association between daily PM_2.5_ concentration and CVD mortality in both *cr* and *tp* spline models. All the smoothing functions of the PCs are statistically significant except for the fourth PC.

**Table 4:** Summary of the GAM-PC analysis for the risk of cerebrovascular disease mortality

	Parametric coefficients		SE			*p* value			
	*cr*	*tp*	*cr*		*tp*	*cr*		*tp*	
Intercept	4.1	4.1	0.012		0.012	< 0.001		< 0.001	
PM_2.5_	1.83 × 10^−5^	3.97 × 10^−5^	1.15 × 10^−4^		1.15 × 10^−4^	0.87		0.73	
DOW1	1.09 × 10^−2^	1.08 × 10^−2^	1.45 × 10^−2^		1.44 × 10^−2^	0.45		0.45	
DOW2	2.93 × 10^−3^	2.42 × 10^−3^	1.44 × 10^−2^		1.45 × 10^−2^	0.84		0.87	
DOW3	1.63 × 10^−2^	1.58 × 10^−2^	1.44 × 10^−2^		1.44 × 10^−2^	0.26		0.27	
DOW4	6.68 × 10^−3^	6.55 × 10^−3^	1.44 × 10^−2^		1.44 × 10^−2^	0.64		0.65	
DOW5	5.62 × 10^−3^	5.53 × 10^−3^	1.44 × 10^−2^		1.44 × 10^−2^	0.70		0.70	
DOW6	− 1.31 × 10^−3^	− 1.31 × 10^−3^	1.44 × 10^−2^		1.45 × 10^−2^	0.93		0.92	
The approximate significance of smoothing terms									
	Edf		Ref.df		Chi-square		*p* value		
	*cr*	*tp*	*cr*	*tp*	*cr*	*tp*	*cr*	*tp*	
s(PC1)	2.77	2.69	2.96	2.93	54.28	50.98	< 0.001	< 0.001	
s(PC2)	1	1	1	1	7.22	6.04	0.007	0.01	
s(PC3)	1	1	1	1	8.06	8.50	0.004	0.003	
s(PC4)	1.55	1.44	1.83	1.74	1.40	0.50	0.43	0.63	
s(t)	17.21	18.05	17.91	18.87	579.74	577.9	< 0.001	< 0.001	
*R*-sq.(adj)		Deviance explained		REML		Scale estimate		*N*	
*cr*	*tp*	*cr*	*tp*	*cr*	*tp*	*cr*	*tp*	*cr*	*tp*
0.637	0.637	63.9%	64%	3990.6	3998.3	1	1	1091	1091

*DOW*, the day of the week; *PC*, principal component; *SE*, standard error; *s()*, smoothing function; *t*, time; *cr*, cubic regression; *tp*, thin plate; *Edf*, effective degrees of freedom; *Ref.def*, reference degrees of freedom; *REML*, restricted maximum likelihood; *N*, total number of observations. Parameter coefficients are adjusted estimates

### Results of the GAM shrinkage smoothers approach

Both shrinkage smoothers (*cr* and *tp*) shrank daily average atmospheric pressure, maximum atmospheric pressure, minimum atmospheric pressure, minimum temperature, average humidity, average rain volume, average wind speed, maximum wind speed, extreme wind speed, and sunshine to zero (Table 5). Again, no statistically significant association was found between daily PM_2.5_ concentration and CVD mortality after adjusting for meteorological conditions and day of the week. The smoothing terms of average and maximum temperature and daily minimum humidity retained statistically significant.

**Table 5:** Summary of the GAM with shrinkage smoothers analysis for the risk of cerebrovascular disease mortality

		Parametric coefficients			SE				*p* value	
		*cr*	*tp*		*cr*		*tp*		*cr*	*tp*
Intercept		4.1	4.1		0.012		0.012		< 0.001	< 0.001
PM_2.5_		6.9 × 10^−5^	1.5 × 10^−4^		1.1 × 10^−4^		1.2 × 10^−4^		0.52	0.16
DOW1		1.0 × 10^−2^	9.2 × 10^−3^		1.44 × 10^−2^		1.44 × 10^−2^		0.47	0.52
DOW2		4.1 × 10^−3^	3.1 × 10^−3^		1.44 × 10^−2^		1.44 × 10^−2^		0.78	0.83
DOW3		1.9 × 10^−2^	1.7 × 10^−2^		1.44 × 10^−2^		1.44 × 10^−2^		0.20	0.23
DOW4		7.0 × 10^−3^	6.1 × 10^−3^		1.45 × 10^−2^		1.44 × 10^−2^		0.63	0.68
DOW5		5.6 × 10^−3^	4.5 × 10^−3^		1.44 × 10^−2^		1.44 × 10^−2^		0.70	0.75
DOW6		− 1.4 × 10^−3^	− 2.1 × 10^−3^		1.44 × 10^−2^		1.44 × 10^−2^		0.92	0.88
The approximate significance of smoothing terms										
		Edf		Ref.df		Chi-square			*p* value	
		*cr*	*tp*	*cr*	*tp*	*cr*	*tp*		*cr*	*tp*
s(PreAvg)		6.6 × 10^−4^	2.3 × 10^−4^	9	9	0	0		0.54	0.42
s(PreMax)		1.3 × 10^−3^	0.707	9	9	0.001	2.11		0.35	0.08
s(PreMin)		6.9 × 10^−4^	2.2 × 10^−4^	9	9	0	0		0.68	0.85
s(TemAvg)		2.48	4.40	9	9	8.07	49.83		0.005	< 0.001
s(TemMax)		4.11	1.29	9	9	17.55	10.37		< 0.001	< 0.001
s(TemMin)		6.8 × 10^−4^	3.2 × 10^−4^	9	9	0	0.001		0.73	0.38
s(HumAvg)		5.2 × 10^−5^	0.68	9	9	0	1.92		0.31	0.067
s(HumMin)		1.64	1.06	9	9	14.14	9.48		< 0.001	< 0.001
s(Rainvol)		9.5 × 10^−4^	2.5 × 10^−4^	9	9	0	0		0.91	0.91
s(Windspd)		4.4 × 10^−4^	2.3 × 10^−4^	9	9	0	0		0.63	0.87
s(WindMax)		1.0 × 10^−3^	6.5 × 10^−3^	9	9	0.001	0		0.39	0.59
s(WindExt)		4.4 × 10^−4^	6.0 × 10^−4^	9	9	0	0		0.84	0.75
s(Sunshine)		5.1 × 10^−4^	2.3 × 10^−4^	9	9	0	0		0.82	0.82
s(t)		8.94	8.94	9	9	369.12	329.99		< 0.001	< 0.001
*R*-sq.(adj)		Deviance explained		REML			Scale estimate		*N*	
*cr*	*tp*	*cr*	*tp*	*cr*	*tp*		*cr*	*tp*	*cr*	*tp*
0.624	0.616	62.7%	62.1%	4000.7	4014.9		1	1	1091	1091

*DOW*, the day of the week; *SE*, standard error; *cr*, cubic regression; *tp*, thin plate; *s()*, smoothing function; *Edf*, effective degrees of freedom; *Ref.def*, reference degrees of freedom; *REML*, restricted maximum likelihood; *N*, the total number of observations. Parameter coefficients are adjusted estimates

### Results of the GAM-LASSO approach

Daily average temperature and minimum temperature were selected using the LASSO procedure and included in the model (Table 6). The association between daily PM_2.5_ concentration and cerebrovascular mortality became no longer statistically significant after adjustment, but the smoothing term of average temperature retained statistically significant.

**Table 6:** Summary of the GAM analysis with selected variables by LASSO

	Parametric coefficients	SE	*p* value	
Intercept	4.11	0.012	< 0.001	
PM_2.5_	3 × 10^−5^	1.1 × 10^−4^	0.80	
DOW1	9.8 × 10^−3^	0.0144	0.50	
DOW2	2.6 × 10^−3^	0.0144	0.86	
DOW3	0.017	0.0144	0.25	
DOW4	4.6 × 10^−13^	0.0145	0.75	
DOW5	0.0041	0.0144	0.78	
DOW6	− 0.0032	0.0145	0.82	
The approximate significance of smoothing terms				
	Edf	Ref.df	Chi-square	*p* value
s(PressMax)	2.39	3.09	3.98	0.26
s(TemAvg)	4.17	5.28	34.25	< 0.001
s(TemMin)	1.02	1.03	0.32	0.59
s(t)	17.18	17.89	464.62	< 0.001

*DOW*, the day of the week; *SE*, standard error; *s()*, smoothing function; *Edf*, effective degrees of freedom; *Ref.def*, reference degrees of freedom

## Discussion

In the current study, we evaluated the effect of daily PM_2.5_ concentrations on the CVD deaths in Shanghai, China, using GAM analysis with three different approaches for controlling for collinear confounding variables while simultaneously taking into account the linear and nonlinear relationships between meteorological confounding variables and the number of deaths. The daily average PM_2.5_ concentration level in our data was 55 μg/m^3^, and this was much higher than the recommended levels for PM_2.5_ yearly average below 10 μg/m^3^ or 25 μg/m^3^ by the World Health Organization or the European Union, respectively (Thunis et al. 2017). There was a 1.7% elevated risk for CVD death per 10 μg/m^3^ PM_2.5_ concentration in the unadjusted model, but after adjustment for meteorological variables and temporal trend, the exposure to PM_2.5_ was no longer associated. The results were consistent in the three modelling techniques we used.

A body of research has shown that an increase in PM_2.5_ was linked to an elevated risk of stroke (Franklin et al. 2008; Leiva et al. 2013; Lin et al. 2017; Lisabeth et al. 2008; Shah et al. 2015; Wellenius et al. 2012), ischemic heart disease (Pope et al. 2002), myocardial infarction (Peters et al. 2001), and cerebrovascular mortality (Gutierrez-Avila et al. 2018), and recent reviews showed that PM_2.5_ was associated with approximately 1% elevated risk of cerebrovascular mortality by every 10 μg/m^3^ increase (Shah et al. 2015; Wang et al. 2014). These studies revealed an increased risk of CVD associated with short-term exposure to PM_2.5_ in Europe, the USA, Asia, Africa, and South America. The excessive risk per 10 μg/m^3^ increase in PM_2_._5_ concentration ranged from 0.7% in mortality to 1.3% in hospital admission. In our data, the unadjusted association was similar but higher (1.7% excessive mortality per 10 μg/m^3^ increase in PM_2.5_ concentration). It has been proved difficult to quantify premature mortality related to air pollution, notably in regions where air quality is not systematically monitored, and also because the toxic particles from various sources may vary (Tuomisto et al. 2008). The estimated effect of PM_2.5_ on premature mortality largely depends on the toxicity regarding the inhaled particle components. In China, emissions from residential energy use such as heating and cooking have the largest contribution to PM_2.5_, whereas in much of the USA and in a few other countries, emissions from traffic and power generation are important. In the eastern United States, Europe, Russia, and East Asia, agricultural emissions make the largest relative contribution to PM_2.5_ (Lelieveld et al. 2015). It might partially explain the difference in the findings between our study and other studies. On the other hand, the difference might be in part due to the profoundly different demographic characteristics, socioeconomic status, or environmental conditions. For example, Shanghai is a megacity with a dense population and high temperature and humidity all the year round, and PM_2.5_ may impact public health differently from other studied areas. Like the aforementioned studies, our study also controlled for the temporal trend of deaths and confounding from meteorological variables. However, we notice that when we adjusted for quite a few meteorological variables, the association became no longer statistically significant. The results were consistent across different modelling strategies, while the nonlinear association of CVD mortality with the meteorological variables retained. The observed PM_2.5_ concentrations in Shanghai were substantially higher than those in Europe or the USA where most of the current studies have been conducted and conclusions derived. The high ambient PM_2.5_ concentration level in itself might mask triggering effect of exposure, and the meteorological factors were only contributing to exacerbation of a PM_2.5_ exposure effect, which might be a potential reason that no statistically significant effect was observed for PM_2.5_ and deserves further investigation in the comparative studies using data from regions with low PM_2.5_ pollution..

The strengths of our study include, first, the adjustment using the rich information of meteorological variables enabled us for detailed control for weather conditions. Weather conditions and time-varying risk factors such as days of the week may cause a significant modification on PM_2.5_ levels and cerebrovascular events (Zhang et al. 2014). Second, multicollinearity among meteorological variables was handled using different approaches of PCA, shrinkage smoothers, and LASSO regularization, and the results were consistent in all the methods. Third, the use of two types of smoothing splines for the GAM model allowed us to compare results to minimize the bias from spline selection, and the results were, again, consistent regardless of types of smoothing splines. In the multicollinearity context, shrinkage methods, such as ridge regression, may reduce the dimensionality of the data by shrinking some coefficient estimates towards zero but not exactly to zero. While in LASSO, one of the correlated coefficients is usually zeroed and the other is assigned the entire impact. Because of this, ridge regression is expected to work better if there are many large parameters of about the same value. LASSO, on the other hand, is expected to come on top when only a few factors actually have impacts (James et al. 2013)

However, our study also has limitations. First, the relatively shorter time period for the analysis limited us to fully assess the long-term time trend in both PM_2.5_ pollution levels and cerebrovascular disease mortality. Second, only city-level PM_2.5_ concentrations from one air monitoring station were available in our study, but the concentration of PM_2.5_ may differ within the city and change during the day, and people’s location would also not be constant. As a megacity with a population of about 24 million and an area of 6,340 km^2^, Shanghai has vastly different PM_2.5_ concentrations and meteorological conditions across the city. Although deaths from 16 administrative districts in Shanghai were available, only aggregated deaths of the whole city and the PM_2.5_ concentrations, as well as meteorological variables, from a single monitoring station were available in the study. It may mask and obscure the spatial and temporal variability of PM_2.5_ effects at particular exposure hotspots, such as the heat island effect in some parts of the city may exacerbate the effects of PM_2.5_ due to high temperatures that may also affect the outcomes. To overcome the lack of spatial variability in PM_2.5_ concentrations and/or meteorological data, a land use regression approach could be incorporated in the future (Liu et al. 2016). Of course, air pollution and mortality data from multisite would be more helpful for adjusting for the confounding from the spatiotemporal variability in PM_2.5_ concentrations and mortalities. Although many studies relied on air pollution information assuming a constant location of people at, for example, their residential addresses, the quantification of exposure using time and activity patterns of individuals will also enhance the understanding (Reis et al. 2018). Third, cerebrovascular mortality risk may vary by age, sex, and socioeconomic factors, but these characteristics were not controlled for in the current study. However, these characteristics tend to be stable within the city given our relatively short study period, thus confounding is unlikely (Pope et al. 2002). Fourth, we focused on the same day’s effect of a single pollutant and did not include multiple pollutants (Wang et al. 2014) or lagged effects (Gutierrez-Avila et al. 2018). Although it is possible that air pollution may cause death after a certain period of time, a systematic review indicates that the risk appeared hardly different by the inclusion of lags (Shah et al. 2015).

## Conclusion

In this study, we aimed to evaluate the effect of PM_2.5_ pollution on CVD mortality using GAM with three different approaches for controlling for confounding factors. The initially statistically significant 1.65% elevated risk of CVD death was no longer observed after adjusting for a number of meteorological variables. As a large number of people are exposed to air pollution, further analysis using data with various measurement times, periods, and detailed pollutant and exposure profiles would contribute to enhancing the understanding of the impact of PM_2.5_ on human health and its mechanism.
